# Detecting IoT Devices and How They Put Large Heterogeneous Networks at Security Risk

**DOI:** 10.3390/s19194107

**Published:** 2019-09-23

**Authors:** Sharad Agarwal, Pascal Oser, Stefan Lueders

**Affiliations:** 1 CMS Experiment, European Organization for Nuclear Research (CERN), 1211 Geneva, Switzerland; 2 Department of Physics, University of Wisconsin Madison, Madison, WI 53706, USA; 3 CERN Computer Security Team, European Organization for Nuclear Research (CERN), 1211 Geneva, Switzerland; p.oser@cern.ch (P.O.); Stefan.Lueders@cern.ch (S.L.); 4 Institute of Distributed Systems, Ulm University, Helmholtzstraße 16, 89081 Ulm, Germany

**Keywords:** Internet of Things, security, vulnerabilities and protective measures, control network security, operation in multi-user environments, risk assessment

## Abstract

The introduction of the Internet of Things (IoT), i.e., the interconnection of embedded devices over the Internet, has changed the world we live in from the way we measure, make calls, print information and even the way we get energy in our offices or homes. The convenience of IoT products, like closed circuit television (CCTV) cameras, internet protocol (IP) phones, and oscilloscopes, is overwhelming for end users. In parallel, however, security issues have emerged and it is essential for infrastructure providers to assess the associated security risks. In this paper, we propose a novel method to detect IoT devices and identify the manufacturer, device model, and the firmware version currently running on the device using the page source from the web user interface. We performed automatic scans of the large-scale network at the European Organization for Nuclear Research (CERN) to evaluate our approach. Our tools identified 233 IoT devices that fell into eleven distinct device categories and included 49 device models manufactured by 26 vendors from across the world.

## 1. Introduction

The Internet of Things has become the latest trend in today’s world. For 2020, the installed base of Internet of Things devices is forecast to grow to almost 31 billion worldwide [1]. Nowadays, devices like printers, switches, routers, phones, and any other electrical devices are all interconnected to increase the ease of access and maintenance, but at the same time it increases the security risk of being compromised.

IoT devices do not have the traditional host-centric security solutions like antiviruses, firewalls, or any safety feature to detect malware. Instead, they run on certain firmware that is hardware-specific, and each type of device has a different protocol on whose principles it runs. As the IoT devices collect a lot of data, these firmwares should be developed by the manufacturers in a secured style, but is rarely the case. Access to the data collected and stored by these devices can aid criminals to gain a lot of sensitive information, like patients’ healthcare data or video footage of the cameras.

The European Organization for Nuclear Research (CERN), the world’s largest High Energy Physics Laboratory and home to the Large Hadron Collider (LHC), is running a plethora of embedded IoT devices. The users in CERN are from a wide variety of fields, ranging from physicists to all kinds of engineers. CERN hosts four major physics experiments—Compact Muon Solenoid (CMS), Atlas, Alice, and Large Hadron Collider beauty (LHCb)—and many other small experiments, which employ a wide range of IoT devices. To name a few, there are programmable logic controllers (PLCs), arduinos, oscilloscopes, thermometers, cameras, and others that are extensively used to run the experiments and the LHC successfully. Like all other international organizations, CERN also operates a large technical infrastructure consisting of other general purpose IoT devices such as CCTV cameras, printers, and IP phones. It is important to know the security footprint of the IoT hardware that is integrated into its network complex: unknown devices can run on firmware versions that are not updated and use old legacy code, which introduces vulnerabilities. However, to secure a device, we need to first learn all about the device. With CERN as our primary resource, we argue that insecure IoT devices can escalate the security risk inherent in large heterogeneous networks.

**Figure 1:**
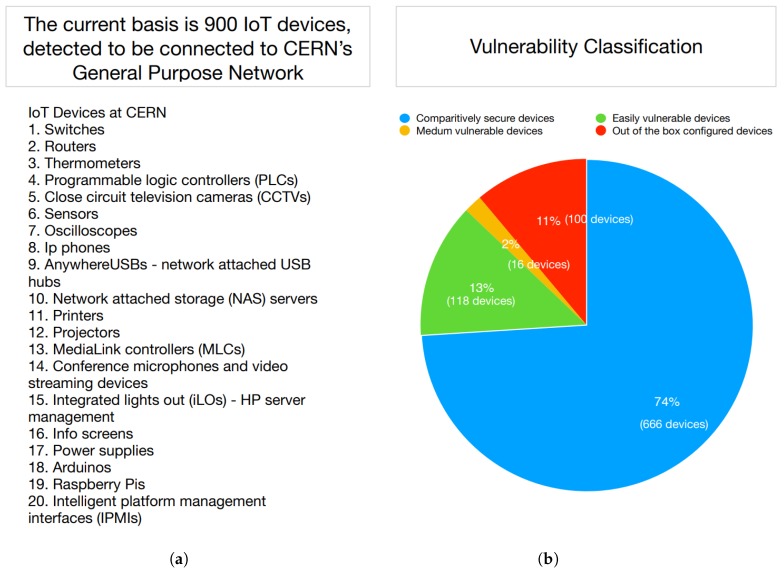
(**a**) Overview of Internet of Things (IoT) devices at the European Organization for Nuclear Research (CERN). (**b**) Vulnerability classification of Internet of Things(IoT) devices at the European Organization for Nuclear Research (CERN).

CERN provides easy network access and allows users to set up and register devices that might help them in their work. Users set up their devices on the CERN network by only registering the media access control (MAC) address of their devices. As the network database does not provide sufficient information to identify and differentiate different IoT devices running on the network, the first step we took was to identify the devices installed at CERN and then did a manual security assessment. As shown in Figure 1b, we classify the identified IoT devices into four vulnerability classes and adapt this paradigm to the CERN network. As IoT devices are networked, they are attractive targets and may become the weakest link for breaking into a secure infrastructure [2], or instead leak sensitive information [3] about users and their behaviors [4]. Integrating these unsecured IoT devices in mission-critical networks with industrial control systems, may put directly controlled assets at risk and possibly endanger the whole connected facility. Earlier this year, the European Union Agency for Network and Information Security (ENISA) published guidelines for the development or repositioning of standards, facilitating the adoption of standards and governance of European Union (EU) standardization in the area of Network Information Security (NIS) [5], but the manufacturers, consumers, and the EU Authorities have not yet fully implemented it.

In this paper, we identify and present a security assessment of 20 categories of various devices connected to the CERN network, as shown in Figure 1. These 20 categories, mentioned in Figure 1a, were identified by us using our “NetScanIoT” tool. The vulnerability classification in Figure 1b was also done by us as per our Vulnerability Assessment results, discussed later in the paper. We not only detected unprotected ports that allow changing the device’s configuration, but also the devices that are prone to remote code execution. Remote code execution can be used as a gateway for an attacker to gain access to the internal network from the outside and dig further while operating on a trustworthy device.

### 1.1. Contributions

In this paper, we present an approach to scan the large heterogeneous network without causing faults on remote devices and identify IoT device models based on the web interface. Installing or modifying anything on the device under test (DUT) is not needed. We list our contributions as follows.

- We developed two tools: “NetScanIoT" tool and “Web-IoT Detection (WID)” tool;
  The “NetSanIoT” tool detects IoT devices on a large heterogeneous network and is able to detect 20 categories of IoT devices;
  The “Web-IoT Detection (WID)” tool identifies the manufacturer name, model, and firmware versions of the respective IoT device. It is able to identify 92.45% of IoT device models and 100% of IoT device that have a web user interface;
- We implemented a manual security assessment of 20 categories of devices that were identified by our “NetScanIoT” tool on the highly heterogeneous, large-scale network at CERN.

None of the devices installed at CERN are CERN-specific or manufactured only for CERN. The devices installed are manufactured by various vendors from across the world, readily available and also used by other organizations and individuals. The next subsection introduces related work and Section 2 explains the methodologies used for detection of IoT devices and the vulnerability assessment. Section 3 describes the evaluation of the “Web-IoT Detection” (WID) approach following the results of the vulnerability assessment, before we discuss our findings.

### 1.2. Related Work

Most of the related work has identified very few unique categories of IoT devices by scanning a network. Scanning can be done either actively or passively. Active scanning is one-to-one probing communication and passive is where the client listens to every channel’s transmission, which is monitored periodically. Some tools also employ web interface fingerprinting but have assumptions and constraints like working on only single-page applications or analyzing the Hyper Text Transfer Protocol (HTTP) response messages only [6]. Other tools depend on Nmap [7] port scanning and downloading the landing page using Curl [8] to find the firmware version for the IoT devices [9], which does not work for all IoT devices. An IP-based IoT Device Detection approach requires the knowledge of servers run by the manufacturers and are able to evaluate using only ten device models by seven vendors [10]. Another solution, “IoTScanner”, detects, by passive measurement, the identifying devices using the packet’s MAC address [11]. We cannot use passive scanning in our tool as we have 1000s of star points at CERN and depending only on MAC address is not sufficiently reliable.

## 2. Materials and Methods

This section introduces the different approaches we developed to detect and identify IoT devices and the vulnerability assessment performed by us. The first subsection explains the tools we developed and the next subsection tells about the vulnerability assessment we tried manually on these identified IoT devices.

### 2.1. Identification

Although all devices connected to CERN’s networks need to be registered, CERN does not have a specific database for IoT devices in particular. There are hundreds of devices running on various networks and new devices being installed every day. CERN provides a way for all users and visitors to add their devices to the network by just registering the MAC address of the device. The central network database of CERN does not have any other detailed information. Therefore, there is no way to distinguish an IoT device from a computer or a cellphone. As we cannot depend solely on MAC addresses to identify an IoT device, we developed tools to detect and identify these IoT devices. These tools provide more information about the device, which can help the administrators in maintaining the security of these devices. This subsection explains the tools we developed to solve this problem as follows.

#### 2.1.1. NetScanIoT Tool

**Figure 2:**
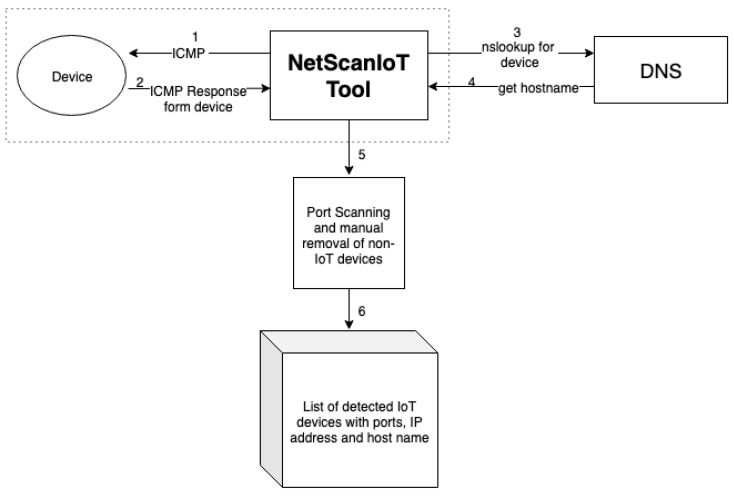
Overview of NetScanIoT tool.

We wrote a Python [12] tool called NetScanIoT, which pings the devices within the network and checks the ICMP [13] message if the target is reachable. If the device response is positive, we go for a nslookup [14] to find the hostname and save the list of the IP addresses along with their hostnames. We prefiltered the output devices by port scanning and then manually also removed the non-IoT devices from the list connected to the network. We were able to identify 20 categories of IoT devices, as shown in Figure 1a. Figure 2 shows a graphical working of the NetScanIot tool. With the help of this tool, we were able to detect 900 physical IoT devices.

#### 2.1.2. Web-IoT Detection (WID) Tool

Scraping a web page can be done with many available tools these days, but with so many different manufacturers, the challenge becomes tough. We initially tried to use Wget [15], Curl, Scrapy [16], and other tools, but there are multiple web pages that require to render JavaScript code, which these tools can not. The reason for doing so is that 20% of the device models’ web pages render JavaScript first to show the complete page source. Therefore, we wrote the WID tool in Python using Selenium [17] in headless mode, which renders the web page with a web driver (Chrome/Firefox) to get the page source. We first analyzed the page source manually and identified six classifiers. With the help of Beautiful Soup [18], we used these classifiers and automated the process to find the category of the IoT device. WID not only scrapes the index page of the device, but also scrapes the sub-URLs recursively, to identify the devices’ information such as the model and the firmware version running on the device.

**Figure 3:**
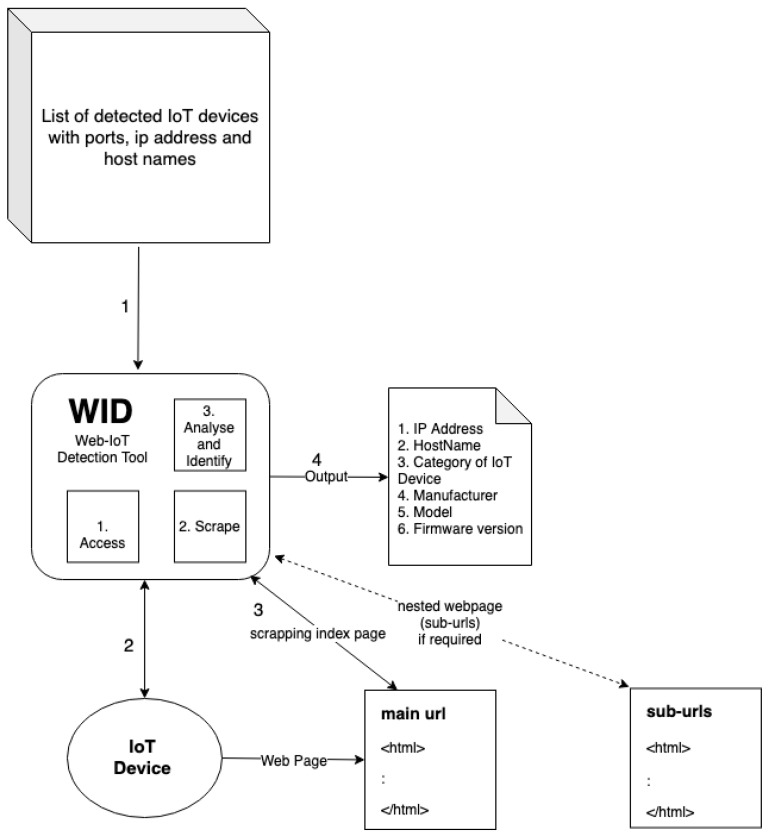
Overview of Web-IoT detection (WID) tool.

**Figure 4:**
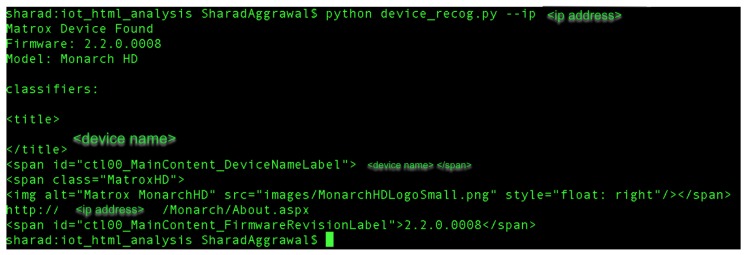
Sample working of Web-IoT detection (WID) tool.

Figure 3 shows an overview of the Web-IoT Detection tool and explains the working of the tool to identify the IoT device. With the help of this tool, we were able to identify 233 physical IoT devices, which have a web interface. Figure 4 shows a sample working of the WID tool that takes an IP address as input and identifies the device, model, and firmware version. We also present the classifiers in the example, which are analyzed from different sub-URLs that the tool scraped.

The WID tool produces the following output.

- IP-address and the host name of the device;
- Web page availability of the device;
- Category of device identified;
- Manufacturer/Vendor name;
- Model name;
- Firmware version.

### 2.2. Vulnerability Assessment

As the Internet of Things is a very new technology, there is no specially designed vulnerability assessment tool that is known to us. There are some general tools like Nessus [19], Open vulnerability assessment system (OpenVAS) [20], and others, but these do not deliver good results, as they do for regular clients or servers. So, we started with the network and web interface of all the IoT devices since it is the primary interface through which the users can connect to the devices. We started looking for web interface injections and attacks to check for the devices. The first step was to use the top Open web application security project (OWASP) IoT Vulnerabilities [21] for investigating the IoT device’s web interface and tried to get administrative access. Our next aim was to find the network side vulnerabilities by scanning the devices and checking the open and filtered ports vulnerable to attack—some of them being secure shell (SSH) [22], Telnet [23], Session initiation protocol (SIP) [24], Real time streaming protocol (RTSP) [25], and JetDirect [26]—and get administrative access into the configurations of the devices. We used software like Printer exploitation toolkit (PRET) [27] and Routerscan [28]. We also modified three available exploits from the Google Hacking Database [29] to find vulnerable IoT devices on the network.

## 3. Results

### 3.1. Evaluation of Tools

In this section, we elaborate on the results of the Web-IoT Detection (WID) tool. As mentioned in Table 1, we show the models and manufacturers names that we are successfully able to identify with our tool. It shows that eleven out of 20 categories of devices that have a web interface were identified. We achieve an accuracy of 92.45% as only 49 out of 53 models were identified by the software using the six classifiers. The “NetScanIoT” and the “Web-IoT Detection” tools can be used in any organization or even by an individual to detect, identify, and use the output information to keep their IoT devices secure.

### 3.2. Vulnerability Assessment Results

After the manual security assessment, we found out that 100 of the 900 devices have the default configuration, and we classify this as “Out of the box configured” category. The devices of this category had no authentication setup on the web user interface or the command-line interface. The “Easily vulnerable” category consisted of 118 devices that had easily guessable or standard manufacturer-configured credentials, which made them easily accessible to users within the network. Apart from this, there were certain devices like thermometers which had a hard-coded super admin password that cannot be changed. We also found 16 devices vulnerable to known exploits, which included Real-Time Streaming Protocol (RTSP) Bypass authentication. This exploit affects two manufacturers’ Close Circuit Television Cameras (CCTV) with various running firmware versions. There is more than one model prone to this exploit. Using the PRET software and the JetDirect port, we were able to access the configuration of printers installed at CERN. Additionally, we also discovered that we are able to change the standard welcome message on most of the printers.

Once the vulnerability assessment was completed, we wanted to mitigate the vulnerabilities by reporting them to the administrators and users of the devices. Sending emails to each and every affected device administrator and user at CERN was a tedious task, so we used a platform called Fast Incidence Response (FIR) [30] modified at CERN according to our needs. FIR is a centralized platform and is used to report devices to their owners and responsible users. We used this to report about the affected devices to the responsible owners and provided them with more information on how to mitigate the issues. By doing this assessment, we raise security awareness at CERN. Adding all devices from the categories, as mentioned in Figure 1b, there were 234 vulnerable devices, which were reported and suggested solutions to mitigate them as that could have caused security issues.

## 4. Discussion

We present our results to identify and assess IoT devices on a large-scale and in a heterogeneous network. The tools developed by us can be used by any organization or an individual, to detect and identify IoT devices. The information provided by the tools can be used to secure their IoT devices. With our NetScanIoT software, a total of 20 categories of IoT devices were identified successfully. After identifying these devices, we performed a manual vulnerability assessment on them. This assessment showed that IoT manufacturers did not secure their devices and, moreover, certain devices, like the thermometers [31], did not even allow the user to change the credentials at all. The Web-IoT detection (WID) tool was able to identify eleven out of 20 categories of IoT devices consisting of 49 various models, manufactured by 26 different vendors from across the world. We also identified the corresponding manufacturer and firmware version for these 49 device models of IoT devices, which can be used for risk identification, associated with these firmware versions. None of these identified devices are CERN-specific or specially manufactured for CERN. They are the same devices that the manufacturers sell in the global market.

One of the significant findings was that 118 devices administered by 90 users were using default passwords and old firmware versions. The administrators did not consider to change them at all as they were not made aware by any kind of prompt that they should change the default password or update to the latest firmware version of the device. Therefore, we propose periodic scans on all networks to detect devices that might be vulnerable.

We showed that the approach is effective on a large-scale network with a larger dataset compared to similar studies. Moreover, no other work was able to classify this amount of heterogeneous IoT device models by using the web interface. For future work, we plan to identify new types of IoT devices that come up together with industrial IoT devices on our accelerator complex testbed.

## 5. Conclusions

The paper provides a novel approach to detect and identify Internet of Things (IoT) devices on a large heterogeneous network. The paper also explains the vulnerability assessment carried out on the large heterogeneous network at CERN and provides results with a vulnerability classification. The “NetScanIoT” tool and the “Web-IoT Detection” (WID) tool can successfully detect and identify IoT devices and also provide more information such as model, manufacturer and firmware version of the device. The tools provide an accuracy of 92.45% for identification of an IoT device. The tools were successfully able to identify 29 IoT device models manufactured by 26 different vendors from across the world.

## Figures and Tables

**Table 1:** Devices detected by WID.

Category	Model	Manufacturer	Quantity	City, Country of Manufacturer
Matrox	Matrox Monarch HD	Matrox	26	Dorval, QC, Canada
Matrox	Matrox LCS	Matrox	2	Dorval, QC, Canada
Telepresence	SX20	Cisco	23	San Jose, CA, USA
Telepresence	C20/40	Cisco	10	San Jose, CA, USA
Oscilloscope	Tektronix	Tektronix	3	Beaverton, OR, USA
Oscilloscope	Lecroy	Teledyne Lecroy	3	New York, USA
Oscilloscope	Keysight53230A	Keysight Technologies	3	Santa Rosa, CA, USA
IP Phone	Polycom	Polycom	2	San Jose, CA, USA
IP Phone	Cisco	Cisco	2	San Jose, CA, USA
IP Phone	FLX	Revolabs	1	Sudbury, MA, USA
IP Phone	Yealink	Yealink	1	Xiamen, China
NAS	Diskstation	Synology	24	Taipei, Taiwan
Printer	Color Laserjet M553	Hewlett Packard	5	Palo Alto, CA, USA
Printer	Laserjet 500 color	Hewlett Packard	4	Palo Alto, CA, USA
Printer	Color Laserjet m750	Hewlett Packard	2	Palo Alto, CA, USA
Printer	Laserjet 2430	Hewlett Packard	3	Palo Alto, CA, USA
Printer	3130 cn	Dell	2	Round Rock, Texas, USA
Printer	DCP-L	Brothers	2	Aichi Prefecture, Japan
Printer	HL-5470	Brothers	1	Aichi Prefecture, Japan
Printer	HL-3070CW	Brothers	1	Aichi Prefecture, Japan
Printer	mfc-8370 dn	Brothers	1	Aichi Prefecture, Japan
Printer	Color Laserjet mfp m277	Hewlett Packard	3	Palo Alto, CA, USA
Printer	Laserjet cp1525N	Hewlett Packard	1	Palo Alto, CA, USA
Printer	Color Laserjet cm1312nfi mfp	Hewlett Packard	1	Palo Alto, CA, USA
Printer	Laserjet 400 m401	Hewlett Packard	1	Palo Alto, CA, USA
Printer	Star Asura	Star POS Printing Soln.	3	Shizuoka, Japan
Printer	HP envy	Hewlett Packard	3	Palo Alto, CA, USA
Printer	Photosmart plus printer	Hewlett Packard	2	Palo Alto, CA, USA
Printer	Designjet T120	Hewlett Packard	2	Palo Alto, CA, USA
Printer	Epson wf-3720 series	Epson	1	Nagano Prefecture, Japan
Printer	zebra zbr3878142	Zebra	1	Illinois, USA
Printer	sws/syncthru	Samsung	2	Seoul, South Korea
Printer	Officejet pro l7700	Hewlett Packard	1	Palo Alto, CA, USA
Infoscreens	GM F420SEA F470S/GM F420S	JVC	9	Kanagawa Prefecture, Japan
CCTV Camera	cc8370	Vivotek	11	New Taipei City, Taiwan
CCTV Camera	ip8365eh	Vivotek	9	New Taipei City, Taiwan
CCTV Camera	Flexidome ip corner 9000 mp	Bosch	6	Gerlingen, Germany
CCTV Camera	M1114	Axis	2	Lund, Sweden
CCTV Camera	q6000-e	Axis	3	Lund, Sweden
CCTV Camera	P5635-E MKII	Axis	4	Lund, Sweden
CCTV Camera	Q24	Mobotix AG	3	Winnweiler, Germany
CCTV Camera	M24	Mobotix AG	2	Winnweiler, Germany
CCTV Camera	M25	Mobotix AG	6	Winnweiler, Germany
CCTV Camera	DCS-910	D-link	2	Taipei, Taiwan
CCTV Camera	AW-HE60H	Panasonic	2	Osaka Prefecture, Japan
CCTV Camera	SNC-RZ50	Sony	1	Tokyo, Japan
PLC	Saia	SBC	10	Murten, Switzerland
Arduino	Arduino Yun/Uno	Arduino	9	Somerville, MA, USA
IPMI	ILO	Hewlett Packard	12	Palo Alto, CA, USA

## Abbreviations

The following abbreviations are used in this manuscript.

IoT	Internet of Things
WID	Web-IoT Detection
CERN	European Organization for Nuclear Research
LHC	Large Hadron Collider
DUT	Device Under Test
PLC	Programmable Logic Controller
ENISA	European Union Agency for Network and Information Security
NIS	Network Information Security
UI	User Interface
NAS	Network Attached Storage
MLC	Media Layer Controller
IP	Internet Protocol
MAC	Media Access Control
HTTP	Hypertext Transfer Protocol
SSH	Secure Shell
CCTV	Close Circuit Television
OWASP	Open Web Application Security Project
SIP	Session Initiation Protocol
RTSP	Real-Time Streaming Protocol
URL	Uniform Resource Locator
LHCb	Large Hadron Collider beauty
CMS	Compact Muon Solenoid
